# Complete Genome Sequence of Bradyrhizobium japonicum Podophage Paso

**DOI:** 10.1128/MRA.01444-20

**Published:** 2021-02-04

**Authors:** Dylan McBee, Aravind Ravindran, Heather Newkirk, Ben Burrowes, Ry Young, Carlos Gonzalez

**Affiliations:** a Department of Biochemistry and Biophysics, Texas A&M University, College Station, Texas, USA; b Department of Plant Pathology and Microbiology, Texas A&M University, College Station, Texas, USA; c Center for Phage Technology, Texas A&M University, College Station, Texas, USA; Portland State University

## Abstract

Bradyrhizobium japonicum is a nitrogen-fixing, Gram-negative bacterium that forms a symbiotic relationship with leguminous plants. This announcement describes the isolation and genome annotation of B. japonicum T7-like podophage Paso. Genomic analysis reveals genes that are associated with both the T5 and T7 modes of genomic DNA entry into the host.

## Abstract (precis)

Bradyrhizobium japonicum is a nitrogen-fixing, Gram-negative bacterium that forms a symbiotic relationship with leguminous plants. This announcement describes the isolation and genome annotation of B. japonicum T7-like podophage Paso. Genomic analysis reveals genes that are associated with both the T5 and T7 modes of genomic DNA (gDNA) entry into the host.

## ANNOUNCEMENT

Bradyrhizobium japonicum is found in leguminous root tips, where it ultimately stimulates soybean growth (1). The nitrogen-fixing properties of B. japonicum have made it a popular inoculant in crop production, with recent efforts to identify remedial applications (2, 3). Characterizing Paso may accelerate efforts to understand this bacterium and the larger ecosystem that it inhabits.

Phage Paso was isolated by plaque purification (4) from unidentified weed samples from Uvalde, TX, in August 2017 and propagated aerobically on B. japonicum D409 (ATCC 10324) at 28°C in l-arabinose medium. Genomic DNA was purified using a Wizard DNA kit (5). Libraries were prepared with 550-bp inserts using a TruSeq Nano kit. The paired-end 500-bp reads were sequenced on an Illumina MiSeq instrument with v2 500-cycle chemistry. The 1,997,626 reads were quality controlled and trimmed with FastQC (www.bioinformatics.babraham.ac.uk/projects/fastqc) and then FastX v0.0.14 (http://hannonlab.cshl.edu/fastx_toolkit/download.html) before assembly into a single contig at 1,031.0-fold coverage with SPAdes v3.5.0 (6), which was closed based on the circular assembly generated by the assembler. GLIMMER v3 and MetaGeneAnnotator v1.0 structural annotation outputs were manually verified to predict protein-coding genes, while tRNA genes were predicted with ARAGORN v2.36 (7–9). Protein functions were predicted using outputs from BLAST v2.9.0 and InterProScan v5.33 (10, 11). Putative transmembrane domains were detected using TMHMM v2.0 (12). BLAST queries were compared against the NCBI nonredundant, UniProtKB Swiss-Prot, and TrEMBL databases with a 0.001 maximum expectation value cutoff (13). The genome-wide DNA sequence similarity between Paso and other phages was calculated by progressiveMauve v2.4 (14). All annotation tools were accessed via the CPT Galaxy interface hosted at https://cpt.tamu.edu/galaxy-pub (15–17). Unless otherwise stated, all tools were executed using default parameters.

The 47,808-bp DNA genome sequence of Paso has a predicted 95% coding density and a GC content of 55% compared to 64% in the host (1). PhageTerm (18) predicts the genome sequence to start with a T7-like 371-bp direct terminal repeat. Overall, 55 protein-encoding genes were identified, but no tRNA genes were located; all the coding DNA sequences (CDS) are on one strand. The first 1.5 kb of the genome sequence appears to contain no genes.

*Agrobacterium* phage Atu_ph03 is the closest characterized phage to Paso, with 29 of the 55 genes sharing amino acid sequence similarity, although the overall nucleotide sequence similarity is only 14.7%. Genomic analysis and amino acid and nucleotide similarities suggest that Paso is a podophage, which was confirmed visually by transmission electron microscopy (TEM). Interestingly, Paso contains a close homolog of the T5 A1 protein and the T7 RNA polymerase. Analysis by HHpred identified an unusual Clp protease speculated to play a role in capsid degradation (19, 20). Most of the BLASTp hits for Paso genes are from bacterial genomes, suggesting a temperate life cycle for Paso or its close relatives.

### Data availability.

The Paso genome is deposited as GenBank accession number MT708546.1. The associated BioProject, SRA, and BioSample accession numbers are PRJNA222858, SRR11558344, and SAMN14609645, respectively.
